# Reducing Surgery for Pediatric Posttonsillectomy Hemorrhage Using Tranexamic Acid: A Quality Improvement Initiative

**DOI:** 10.1002/ohn.1300

**Published:** 2025-05-21

**Authors:** Laura A. Petrauskas, Janavi Sethurathnam, Ansley J. Kunnath, Rahul K. Sharma, John Ceremsak, Ryan H. Belcher, James D. Phillips, Jay A. Werkhaven, Amy S. Whigham, Lyndy J. Wilcox, Christopher T. Wootten, Frank W. Virgin, Jason S. Park

**Affiliations:** ^1^ Department of Otolaryngology Louisiana State University Health Sciences Center New Orleans Louisiana USA; ^2^ Vanderbilt University School of Medicine Nashville Tennessee USA; ^3^ Department of Otolaryngology–HNS Vanderbilt University Medical Center Nashville Tennessee USA; ^4^ Vanderbilt University Medical Center, Division of Pediatric Otolaryngology–HNS Nashville Tennessee USA

**Keywords:** pediatric otolaryngology, posttonsillectomy hemorrhage, quality improvement, tranexamic acid

## Abstract

**Objective:**

Evaluate the use of tranexamic acid (TXA) and observation as a management option for pediatric patients presenting with posttonsillectomy hemorrhage (PTH).

**Study Design:**

Retrospective analysis of a prospectively implemented quality improvement initiative with a historical control comparison group.

**Setting:**

Tertiary children's hospital.

**Methods:**

Patients <18 years of age who underwent adenotonsillectomy (AT) and returned to the Emergency Department for PTH were included. Patients who were stable without large volume or active bleeding were given intravenous TXA and admitted for overnight observation. Data were compared in a before‐and‐after analysis: preprotocol (April 2022 to March 2023) versus postprotocol (April 2023 to March 2024). For cost‐effectiveness analysis, we analyzed aggregated claims data from a commercial claims database.

**Results:**

Preprotocol 1800 adenotonsillectomies were performed, and 40 procedures were performed for control of hemorrhage (2.2 per 100 AT). Postprotocol 2356 adenotonsillectomies were performed, and 30 procedures were performed to control hemorrhage (1.3 per 100 AT) showing a significant reduction in return to the operating room (relative risk [RR] = 0.59, 95% confidence interval [CI] [0.358, 0.916], *P*‐value .020). There were no reported adverse events attributable to TXA. An estimated 21 surgeries were avoided, and 26 additional patients were observed in the hospital during the postprotocol period, for an estimated net cost savings of $174,970.

**Conclusion:**

The implementation of a standardized TXA protocol significantly reduced the need for return to the operating room for PTH in pediatric patients, without complications and with net cost savings to the healthcare system.

Posttonsillectomy hemorrhage (PTH) remains a common and problematic complication of adenotonsillectomy (AT), which is the second most commonly performed outpatient pediatric surgical procedure in the United States.^1^ Rates of primary and secondary bleeding following tonsillectomy of up to 0.1% to 7% are reported in the literature and can result in significant morbidity and in rare cases, death.^2^, ^3^, ^4^ Typical treatment strategies for PTH include readmission for observation and/or surgery to control bleeding, depending on the severity of presentation and patient factors.

Tranexamic acid (TXA) is an inexpensive antifibrinolytic agent that can be given in an oral, intravenous (IV), topical, or nebulized form. TXA reduces bleeding by competitive inhibition of the activation of plasminogen to plasmin, a molecule responsible for the degradation of fibrin.^5^ Side effects are not common but include headache, nausea, vomiting, dizziness, hypersensitivity reactions, and visual disturbances.^6^ TXA is US Federal Drug Administration (FDA) approved only for heavy menstrual bleeding and for short‐term prevention of bleeding following dental extraction in patients with hemophilia.^6^ However, a PubMed search at the time of the writing of this manuscript will show almost 8000 articles examining many uses of TXA ranging from trauma indications, obstetrics, orthopedics, and more.^7^, ^8^, ^9^ Numerous studies have evaluated the use of TXA in otolaryngology. For example, the role of TXA in epistaxis has been studied with one study demonstrating that topical use of TXA for control of epistaxis as compared to anterior nasal packing showed a significant reduction of bleeding time.^10^ In sinus surgery, a meta‐analysis of seven studies that used perioperative TXA showed decreased operative time, reduction in intraoperative blood loss, and increased satisfaction of surgeons.^11^ Recently, studies have begun to examine the role of TXA in the treatment of PTH. Of particular interest is a retrospective case control study by Smith et al that found patients with PTH who received IV TXA compared to historical controls who did not receive TXA had a statistically significant decreased need for return to the operating room (OR) (*P* = .042).^12^ This was the only study we found that focused on pediatric patients presenting with PTH and using IV TXA as a treatment option.

Before April 2022, management of pediatric PTH in our hospital was based on individual decision‐making and clinical judgment of the surgeon on call. In April 2022, as a quality improvement initiative, we created and prospectively implemented a standardized protocol including the use of IV TXA and observation as a management option for children presenting to the Emergency Department (ED) at our hospital as a quality improvement initiative. Here, we describe the results of this protocol implementation and evaluate its impact on the need for operative intervention for control of PTH. We hypothesized that following the implementation of the TXA protocol, the number of patients who are required to return to the OR for control of hemorrhage would be decreased compared to the previous.

## Methods

This is a 1‐year analysis of outcomes after prospective implementation of a TXA protocol at a tertiary children's hospital, with a historical control comparison group. This study was approved for exemption by the Vanderbilt University Medical Center Institutional Review Board. The study population includes patients <18 years of age who underwent AT and presented to the Vanderbilt Children's Hospital ED with PTH. Per the protocol, on arrival to the ED patients who presented with PTH required IV access if they had active bleeding, recent bleeding, clot, signs of hypovolemia, or if being admitted. Complete blood cell count (CBC) and type and screen (T&S) were obtained for patients with large volume active bleeding, history of large volume bleeding, or multiple bleeds or signs of hypovolemia. Patients who presented with active bleeding, a large clot with continued evidence of oozing, or a clot with a significant bleed history before ED presentation were taken for operative management. Patients who had the presence of a stable clot without signs of hypovolemia, a concerning history for bleeding but normal exam, previous ED presentation with concerns for bleeding, or bleeding history with a normal exam but with poor oral intake or pain control issues were admitted for observation.

All patients who were admitted or going to the OR from the ED were given IV TXA at a dose of 10 mg/kg every 8 hours until discharge. If admitted for observation, the patient was monitored with serial exams while continued on IV TXA and discharged once there was no longer evidence of bleeding and the patient was tolerating a diet and with good pain control, per the surgeon's discretion. On discharge, patients were prescribed oral TXA at a dose of 10 to 30 mg/kg by mouth every 8 hours with a maximum dose of 1300 mg/dose for 5 days. Oral TXA is only readily available in 650 mg tablet formulation in the United States and is approximately 30% to 50% bioavailable; given these points and the favorable safety profile of the medication, we accepted a wide range of oral dosing for simplicity.

Data from patients that returned to the ED within 30 days after AT was performed by pediatric otolaryngologists at Vanderbilt University Medical Center were obtained and included. Patients presenting with PTH whose surgeries were performed at other Vanderbilt Children's sites, outside facilities, or by outside surgeons were excluded to ensure a complete data set. Collected variables included age, sex, body mass index (BMI) race, insurance type, comorbidities (including; obesity, asthma, history of prematurity, trisomy 21, cerebral palsy, sickle cell disease/trait, epilepsy/seizures, craniofacial abnormalities, bleeding disorder, connective tissue disease, and other), indication for AT, whether polysomnogram was obtained, severity of obstructive sleep apnea (OSA), tonsil and adenoid size, method of tonsillectomy, postoperative day for return to ED, whether patients received TXA, whether patients received operative intervention for PTH, and whether patients were discharged on TXA. Study data were collected and managed using REDCap electronic data capture tools hosted at Vanderbilt University. REDCap is a secure HIPAA‐compliant web‐based software platform.^13^, ^14^ Outcomes were studied in a before‐and‐after analysis between two time periods: preprotocol (April 2022 to March 2023) and postprotocol (April 2023 to March 2024). All analyses were conducted in R (R Studio PBC). Pearson's chi‐square test, Fisher's exact test, and Mann‐Whitney *U* tests were used to evaluate differences in clinical outcomes between preprotocol and postprotocol time periods. The relative risk (RR) was derived as the ratio of the surgical procedure rates between the postprotocol and preprotocol groups, and its statistical significance was assessed using a *z*‐test. In addition, binomial logistic regression was calculated to compare patients that did or did not require surgical intervention in preprotocol and postprotocol groups. The primary outcome was returning to the OR after presenting to the ED for PTH. Ancillary outcomes included observation in the ED, overnight admissions, length of stay, and additional ED visits within 30 days after surgery. Data from an aggregated commercial claims database (Marketscan) were used for a cost‐effectiveness calculation. Specifically, all database claims for PTH that presented to the ED were aggregated, and cost data associated with these visits were extracted to understand the average difference in price between those who went to the OR versus those who were discharged without operative intervention. Statistical significance was set at *P* < .05. Run charts were used to track various process and outcome quality improvement metrics.

## Results

The records of 170 unique patients with a return visit to the ED for PTH were reviewed. There were a total of 187 ED visits. Table 1 illustrates the patient characteristics of those who presented with PTH. The median age was 8.1 (range 1.7‐18.4) with most patients being white (N = 112, 66%) and male (N = 86, 51%). Most commonly, patients were of a healthy weight (N = 77, 45%). In total, 41% of patients presenting with PTH carried a diagnosis of recurrent acute tonsillitis. Nearly all patients (97%) received a “hot” tonsillectomy (ie, Bovie monopolar electrosurgery). There were no statistically significant differences in age, sex, race, or BMI between the preprotocol and postprotocol groups.

**Table 1:** Demographic and Clinical Characteristics of the 170 Patients Who Returned to the Emergency Department for Posttonsillectomy Hemorrhage Within the Study Period

Characteristic	N = 170 patients	Preprotocol (N = 78)	Postprotocol (N = 92)	*P*‐value
Age	8.1 (1.7‐18.4)^a^	7.4 (1.7‐18.4)	8.6 (1.8‐17.0)	.012
Sex				.446
Female	84 (49%)	36 (46%)	48 (52%)	
Male	86 (51%)	42 (54%)	44 (48%)	
Race				.086
White	112 (66%)	48 (62%)	64 (69.6%)	
Black	26 (15%)	18 (23%)	8 (8.7%)	
Asian	4 (2%)	1 (1.3%)	3 (3.3%)	
Hispanic/Latino	34 (20%)	13 (16.7%)	21 (23%)	
Unknown	1 (0.6%)	0	1 (1.1%)	
Other	3 (1.8%)	1 (1.3%)	2 (2%)	
BMI				.088
Underweight	13 (8%)	6 (8%)	7 (7.6%)	
Healthy weight	77 (45%)	32 (41%)	45 (49%)	
Overweight	27 (16%)	12 (15%)	15 (16.3%)	
Obese	36 (21%)	23 (29%)	13 (14%)	
Severely obese	14 (8%)	3 (4%)	11 (12%)	
Unknown	3 (2%)	2 (3%)	1 (1.1%)	
Indication				
Sleep‐disordered breathing	98 (58%)	49 (63%)	49 (53%)	
OSA	61 (36%)	29 (37%)	32 (35%)	
Recurrent tonsillitis	70 (41%)	37 (47%)	33 (36%)	
Other	83 (39%)	58 (74%)	25 (27%)	
Method				
Cold	3 (1.8%)	2 (3%)	1 (1.1%)	
Intracapsular	2 (1.2%)	1 (1.3%)	1 (1.1%)	
Hot	165 (97%)	75 (96%)	90 (98%)	
Comorbidities				
Obesity	50 (29%)	25 (32%)	25 (27%)	.487
Asthma	16 (9.4%)	8 (10%)	8 (8.7%)	.728
Prematurity	12 (7%)	5 (6%)	7 (7.6%)	.761
Trisomy 21	0	0	0	‐
Cerebral palsy	2 (1.1%)	1 (1.2%)	1 (1.1%)	.906
Sickle cell disease/trait	0	0	0	‐
Epilepsy/seizures	5 (3%)	0	5 (5.4%)	.036
Craniofacial	5 (3%)	2 (2.6%)	3 (3.3%)	.789
Bleeding disorder	1 (0.5%)	1 (1.2%)	0	.276
Connective tissue disorder	1 (0.5%)	1 (1.2%)	0	.276

Abbreviations: BMI, body mass index; OSA, obstructive sleep apnea.

^a^ Mean, range.

Preprotocol 1800 TA were performed, and 40 surgical procedures were performed for control of hemorrhage (2.2 per 100 AT). Postprotocol 2356 TA were performed, and 30 surgical procedures were performed for control of hemorrhage (1.3 per 100 AT), showing a significant reduction in return to OR (RR = 0.59, 95% confidence interval [CI] [0.358, 0.916], *P*‐value .020). There were no reported adverse events attributable to TXA.

**Figure 1:**
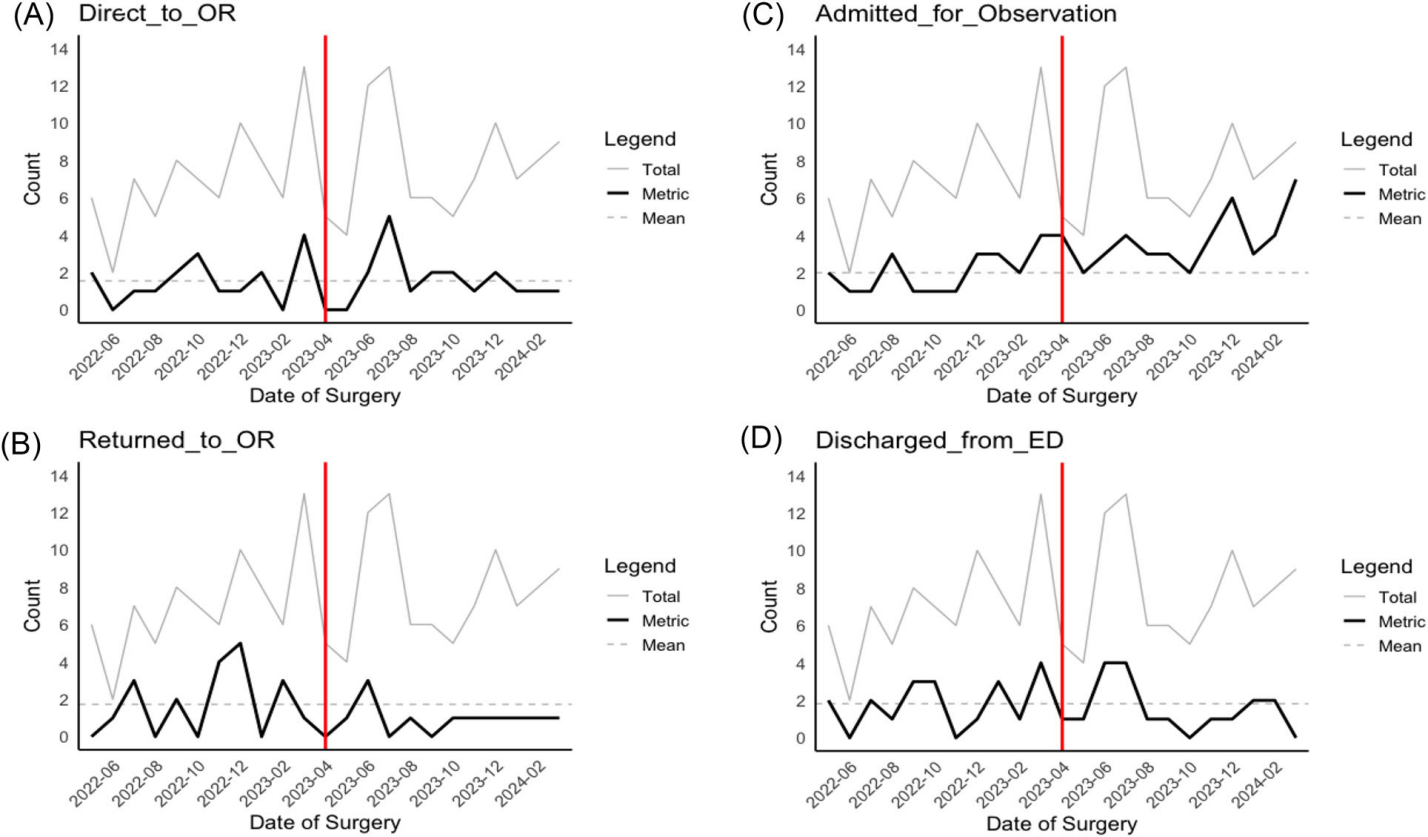
Run charts comparing encounters: (A) direct to operating room (OR), (B) to OR from observation, (C) admitted for observation, and (D) discharged from Emergency Department (ED). Dotted lines indicate the mean value for the preprotocol period. Gray line indicates the total number of encounters for posttonsillectomy hemorrhage that month.

A run chart was generated to track the outcome of the quality improvement protocol change. Figure 1 compares the different patient dispositions that occurred month to month both preprotocol and postprotocol. The run charts show a decrease in patients that required return to the OR following protocol implementation. Additionally, the run chart shows an increase in patients who were admitted for observation post protocol.

**Figure 2:**
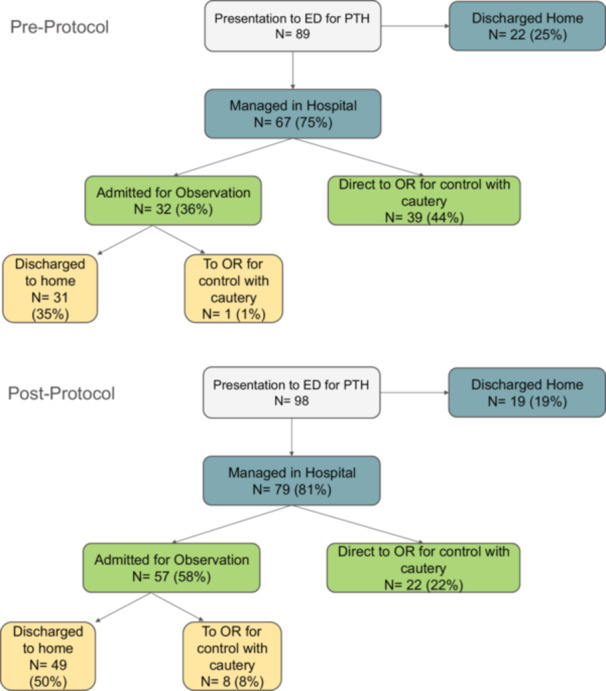
Postoperative Emergency Department (ED) visits for posttonsillectomy hemorrhage (PTH) preprotocol and postprotocol total number of visits, number of patients that were discharged home, those that were managed in the hospital either via observation or directly to the operating room (OR).

The number of patients that returned to the ED for PTH was further examined and demonstrated in Table 2, Figure 2. A total of 187 ED visits were included for analysis. Within the preprotocol period, 40 (45%) of patient encounters required operative intervention for PTH compared to 30 (31%) postprotocol. A similar number of patient encounters resulted in discharge to home in 25% preprotocol as compared to 19% postprotocol. More patients were observed in the hospital postprotocol (58%) than preprotocol (36%). In total, 8 (14%) patients were taken to the OR out of 57 patients who were initially admitted for observation and started on TXA. When comparing patients who went from inpatient observation to the OR, preprotocol, there was one patient as compared to eight in the postprotocol group. There was a statistically significant change in the distribution of outcomes (disposition) between the preprotocol and postprotocol periods (Pearson's chi‐square test, *P* = .003). We then compared the frequency with which encounters resulted in surgical intervention between the preprotocol and postprotocol periods using a binomial logistic regression, which demonstrated a statistically significant decrease in surgery postprotocol (OR = 0.54, 95% CI: 0.30‐0.98; *P*‐value .044). Additionally, we studied the outcomes of patients who were taken directly to the OR from the ED. There were 39 such patients preprotocol. In total, 3 (8%) of these patients later returned to the ED at least once within 30 days for additional bleeding, and 2 of them required repeat surgical management. The other 49 patients (86%) were discharged home, with 2 patients subsequently returning to the ED within 1 week for repeat bleeding and taken to the OR for control of bleeding (Table 3). Hospital length of stay was also not significantly different between the two groups (*P* = .280).

**Table 2:** Outcomes of Patients Presenting to Emergency Department (ED) Within 30 Days Post‐op for Hemorrhage After T&A, Preprotocol and Postprotocol

	Total number of postoperative ED visits for hemorrhage after adenotonsillectomy		
	Preprotocol, N = 89	Postprotocol, N = 98	*P*‐value^a^
Disposition			
ED to discharge	18 (20%)	19 (19%)	.003^a^
Direct to OR	39 (44%)	22 (22%)	
Observation to OR	1 (1%)	8 (8%)	
Observation to discharge	31 (35%)	49 (50%)	
Postoperative day (POD) at presentation			
POD 0	1 (1%)	2 (2%)	.58^b^
POD 1	13 (15%)	10 (10%)	
POD ≥ 2	75 (84%)	86 (88%)	
Hospital stay length in days			
Mean (SD)	1.2 (0.98)	1.3 (0.91)	.28^c^
0	22 (25%)	25 (26%)	
1	37 (42%)	25 (26%)	
2	24 (27%)	45 (46%)	
3	4 (4%)	2 (2%)	
4	1 (1%)	1 (1%)	
5	1 (1%)	0 (0%)	

Abbreviation: OR, operating room.

^a^ Pearson's chi‐square test.

^b^ Fisher's exact test.

^c^ Mann‐Whitney *U* test.

**Table 3:** Analysis of Patients With Additional Return to Emergency Department (ED) for Bleeding After Previous Discharge for Posttonsillectomy Hemorrhage (PTH)

	Preprotocol (N = 78)	Postprotocol (N = 92)	*P*‐value^a^
Return to ED for bleeding after discharge for PTH	7 (9%)	6 (6.5%)	.52
Received surgery during return encounter for management	5 (6%)	2 (2%)	.16
Admitted for observation/discharged home	2 (2.6%)	4 (4%)	.55
Management at prior (first) ED visit			
Discharge home from ED	3 (4%)	1 (1%)	.23
Direct to OR	3 (4%)	3 (3%)	.81
Observation than discharge	1 (1%)	2 (2%)	.67

Abbreviation: OR, operating room.

^a^ Chi‐square test.

**Figure 3:**
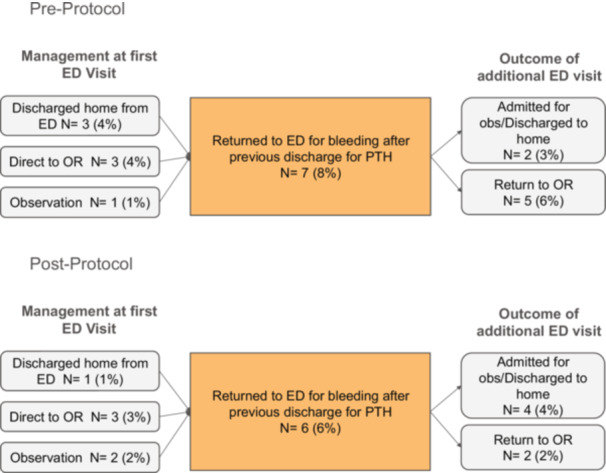
Treatment of patients who had repeat visits to the Emergency Department (ED) for bleeding after discharge for posttonsillectomy hemorrhage (PTH). Preprotocol and postprotocol number of visits, clinical course at first ED visit, and outcome of repeat ED visit for PTH. OR, operating room.

Additionally, patients who returned to the ED again after their initial ED visit for PTH were further analyzed (Figure 3, Table 3). There was no statistically significant difference in the number of patients returning to ED within 30 days of their first ED visit: 7 (9%) preprotocol versus 6 (6%) postprotocol (*P* = .52). Of those additional ED visits, 5 (6%) preprotocol and 2 (2%) postprotocol required additional surgical management. Further breakdown of the seven additional ED encounters preprotocol showed that 3 (4%) had been previously discharged to home, 3 (4%) had previously gone directly to the OR, and 1 (1%) had been admitted for observation. Postprotocol, six additional ED encounters occurred; of those, 1 (1%) had been previously discharged to home, 3 (3%) had gone directly to the OR, and 2 (2%) had been admitted for observation.

We then performed a cost‐effectiveness analysis by extrapolating the number of surgeries that were avoided and the number of additional patients that were observed in the preprotocol period. An estimated 21 surgeries were avoided (2356 × (2.2% − 1.3%)) and an additional 26 patients were observed (98 × (58% − 31%)) in the postprotocol period than would be expected from the preprotocol period. Based on cost estimates of $8253 for return to OR versus $1492 for observation alone (from an aggregated commercial claims database), the estimated net cost savings in the postprotocol period was $174,970 [($8253 × (2356 × (2.2% −1.3%))) − ($1492 × (98 × (58% −31%)))].

## Discussion

To our knowledge, this is the first study to retrospectively analyze a prospectively initiated protocol that assesses the role of IV TXA in reducing the frequency of operative intervention in patients presenting to the ED with PTH in the United States. Administration of TXA as a treatment option for patients presenting with PTH to the ED is a potentially high‐impact and low difficulty intervention that can reduce the need for return to the OR for control of bleeding, making it an attractive intervention to study for quality improvement projects. Over the entire study period, we noted a PTH rate of 2.1%, broadly in line with previous literature in which PTH rates are noted to range from 1% to 7%.^2^, ^3^, ^4^ Our findings show that there was a significant reduction in return to the OR for control of bleeding preprotocol and postprotocol (*P* = .043). Additionally, more patients were admitted for observation postprotocol without a difference in length of stay. Given that the protocol states that if patients are not actively bleeding and are otherwise stable, they should be admitted for observation, it is logical that postprotocol more patients were observed. Additionally as more patients are observed instead of going directly to the OR, we also expect that some of those that are observed would require operative intervention rather than go to the OR to begin with. On further analysis, it was interesting to note that of those patients that were initially admitted for observation and started on the TXA protocol (N = 57), only 8 (14%) required control of hemorrhage in the OR, and 49 patients (86%) were discharged to home. Of those two returned to the OR within 1 week for further bleeding and were taken to the OR for hemorrhage control. This demonstrates that most of the patients who were admitted for observation did not require operative intervention.

Many surgeons believe that primary bleeds (occurring less than 24 hours after surgery) are more severe and of larger volume than secondary bleeds (occurring greater than 24 hours after surgery). To further investigate this thought and examine the effect of TXA on early versus late PTH, we reviewed our data. We found that there were 12 patients who presented with early PTH, and of those 6 (50%) required operative intervention. In the late PTH group, there were 61 patients total, and 21 (34%) required operative control of hemorrhage (*P*‐value = .463) The higher rate of patients in the early PTH group that required OR control of hemorrhage is consistent with the possibility that early bleeds may be more severe or related to vasospasm or technical error and are more likely to require operative intervention despite TXA.

With respect to the therapeutic use of TXA in the management of PTH, the existing literature is predominantly retrospective and single‐institution in nature and varies with respect to the route of administration of TXA. Most of this research has focused on the use of nebulized (rather than IV) TXA. Erwin et al performed a 3‐year retrospective study of patients presenting with PTH. Of the 58 patients included in their study, 14 were given nebulized TXA and 4 (29%) ultimately required operative intervention, compared to 73% of patients in the untreated group (*P* < .05).^15^ Similarly, Shin et al performed a 7‐year retrospective study including adults and children and found that 30 of 83 patients treated with nebulized TXA required operative intervention (36%) compared to 60% in the untreated group (*P* < .05).^16^ Further, Cao et al and Dermendjieva et al contributed case series reports suggesting a possible role for nebulized TXA in the management of PTH.^17^, ^18^

At least two studies have evaluated the role of IV TXA in the management of PTH. Spencer et al performed a 2‐year retrospective study notable in which patients were given either nebulized, IV, or topical TXA. The authors found a significant reduction in the need for operative intervention in those who had received TXA (22% vs 54%, *P* = .026); however, they found no association between the route of TXA administration and need for operative intervention, although the size of each group was relatively small.^19^ Smith et al provided perhaps the strongest evidence for the role of IV TXA in PTH. They performed a large retrospective review in which 195 patients with PTH received 10 mg/kg IV TXA every 8 hours and were observed for 24 hours. Twenty‐six (13%) of these patients required operative intervention compared to 23% of the untreated group (*P* = .042).^12^

Overall, although the literature does suggest a role for the therapeutic administration of TXA in PTH, it remains unclear if the route of administration has a meaningful impact on these results. We chose to administer IV TXA, mirroring the protocol in Smith et al. Consideration of the biological mechanism of action of TXA also supports systemic use. Plasmin is an enzyme that breaks down the cross‐links between fibrin molecules, the structural support of blood clots. TXA competitively inhibits the conversion of plasminogen to its activated form, plasmin, by binding to lysine‐binding sites; it additionally binds to lysine‐binding sites on plasmin itself.^20^ Plasminogen is produced by the liver and circulates in the bloodstream. From a mechanistic standpoint, we would expect TXA to be more effective when administered intravenously rather than topically on the surface of the postsurgical wound bed via nebulizer. Systemically administered TXA carries a theoretical risk of thrombosis, particularly when administered in a systemic fashion in those who have an intrinsic predisposition for thromboembolic disease or in women using combination hormonal contraception.^21^ Previous studies investigating the safety of systemic TXA across a range of pathologies have identified adverse effects including seizures, pulmonary embolism, venous thromboembolism, and vision changes.^22^, ^23^, ^24^, ^25^ With respect to tonsillectomy in particular, reported side effects associated with TXA administration are minor, with one meta‐analysis noting only a few sporadic instances of dizziness and headache.^26^ We noted no reported adverse events associated with IV TXA administration in our cohort.

There are several limitations to this study's analysis and generalizability. There was no placebo group, and patients were not prospectively followed to elicit adverse events as in a randomized controlled clinical trial. The protocol was performed at a single institution (eight surgeons), which may limit generalizability. Ultimately, the decision to return to the OR for control of hemorrhage was made by each individual clinician. Recurrent bleeding despite TXA was the most common indication for returning to the OR; however, it should be noted that there were no objective criteria dictating the need for operative intervention. There is also a source of bias in that surgeons were not blinded to the administration of TXA, which may have made them more likely to delay operative intervention, allowing for the spontaneous resolution of some bleeds regardless of any pharmacologic effect of the TXA itself. Future randomized controlled trials would be indicated for further investigation.

Following a 1‐year analysis after implementation of a protocol for management of PTH including IV TXA administration, we have found a reduction in the rate of overall return to OR for control of hemorrhage, without complications, and with net cost savings to the healthcare system. We have also identified opportunities for ongoing protocol improvement and clarification. These include ensuring that all patients who were admitted for observation for PTH are discharged with 5 days of TXA and standardization of management during the inpatient observation period including nil per os (NPO) and diet management.

## Implications for Practice

Implementation of a “TXA protocol” standardizes care and may reduce the need for return to the OR for pediatric patients presenting with PTH.

## Author Contributions

**Laura A. Petrauskas**, design, conduct, analysis, manuscript preparation, presentation of research; **Janavi Sethurathnam**, data collection, analysis, manuscript review; **Ansley J. Kunnath**, data collection, analysis, manuscript review; **Rahul K. Sharma**, data analysis, manuscript review; **John Ceremsak**, manuscript preparation and review; **Ryan H. Belcher**, conduction, manuscript review; **James D. Phillips**, conduction, manuscript review; **Jay A. Werkhaven**, conduction, manuscript review; **Amy S. Whigham**, conduction, manuscript review; **Lyndy J. Wilcox**, conduction, manuscript review; **Christopher T. Wootten**, conduction, manuscript review; **Frank W. Virgin**, design of QI, conduction, manuscript review; **Jason S. Park**, design of QI, conduction, analysis, manuscript preparation, manuscript review, oversight of project.

## Disclosures

### Competing interests

There are no conflicts of interest.

### Funding source

None.
